# Supplementary material for: Treatment of Cesarean Scar and Cervical Pregnancies Using the Ovum Aspiration Set for Intrachorial Methotrexate Injection as a Conservative, Fertility-Preserving Procedure
Source: Medicina (Kaunas). 2023 Apr 14;59(4):761. doi: 10.3390/medicina59040761 (PMC10143318; doi:10.3390/medicina59040761)
Supplement: Supplementary file 1 [file medicina-59-00761-s001.zip › medicina-2307569-supplementary.pdf]

Table S1: Factors related with the success/failure of MTX (IC/IM) injection (logistic regression)

Variable	Success*<br>Mean (range)<br>N=8	Failure**<br>Mean (range)<br>N=3	Odds Ratio<br>(95% Confidence interval)	p-value
**Gestation type**				0.90
CSP	3 (75%)	1 (25%)		
CP	5 (71.4%	2 (28.6%)	0.83 (0.05 – 13.63)	
**Maternal age (median)**				0.93
≤ 34 years	3 (50%)	3 (50%)		
> 34 years	5 (100%)	0	>999.9 (<0.001 – >999.9)	
**Gravidity**				0.62
≤ 2	4 (80%)	1 (20%)		
> 2	4 (66.7%)	2 (33.3%)	2.00 (0.13 – 31.98)	
**Parity**				0.78
Nulliparous	2 (66.7%)	1 (33.3%)		
Multiparous	6 (75%)	2 (25%)	0.67 (0.04 – 11.94)	
**Duration since last pregnancy (years)**				0.90
≤ 2	5 (71.4%)	2 (28.6%)		
> 2	3 (75%)	1 (25%)	0.83 (0.05 – 13.63)	
**Gestational age at diagnosis (median)**				0.62
≤ 6 weeks	4 (66.7%)	2 (33.3%)		
> 6 weeks	4 (80%)	1 (20%)	0.50 (0.031 – 7.99)	
**Days between diagnosis and therapy Initiation**				0.40
≤ 3 days	5 (83.3%)	1 (16.7%)		
> 3 days	3 (60%)	2 (40%)	3.33 – 0.20 – 54.53	
**Cycles of IM MTX**				0.90
≤ 1	3 (75%)	1 (25%)		
> 1	5 (62.5%)	2 (66.7%)	1.2 (0.007 – 19.63)	
**ß-hCG (at diagnosis, mlU/ml)**				0.62
≤ 38 820 (median)	4 (66.7%)	2 (33.3%)		
> 38 820	4 (80%)	1 (20%)	0.50 (0.031 – 7.99)	
**ß-hCG (at therapy initiation, mlU/ml)**				0.62

≤ 40 661	5 (83.3%)	1 (16.7%)		
> 40 661	3 (60%)	2 (40%)	2.00 (0.13 – 31.98)	
**Localization of gestational sac according to modified Delphi method**				0.81
Not crossing UL/SL	2 (66.7%)	1 (33.3%)		
Crossing UL	1 (100%)	0 (0%)		
Crossing SL	2 (66.7%)	1 (66.7%)	1.50 (0.06 – 40.63)	
**History of miscarriage**				0.93
No	5 (100%)	0 (0%)		
Yes	3 (50%)	3 (50%)	>999.99 /<0.001 – >999.99)	

(Abbreviations: CSP=cesarean scar pregnancy, CP=cervical pregnancy, HCG=human chorionic gonadotropin, IM=intramuscular, MTX=methotrexate, SL=serosal line, UL=uterine line)
